# ‘I don’t talk about my distress to others; I feel that I have to suffer my problems...’ Voices of Indian women with breast cancer: a qualitative interview study

**DOI:** 10.1007/s00520-020-05756-8

**Published:** 2020-09-21

**Authors:** Sunitha Daniel, Chitra Venkateswaran, Ann Hutchinson, Miriam J. Johnson

**Affiliations:** 1 Department of Palliative Medicine, National Health Mission, General Hospital Ernakulam, Kochi, India; 2 Wolfson Palliative Care Research Centre, Hull York Medical School, Allam Medical Building, University of Hull, Hull, HU6 7RX UK; 3 Department of Psychiatry, Palliative Care and Psycho Oncology, Believers Church Medical College, Thiruvalla, Kerala India

**Keywords:** Breast neoplasms, Stress, Psychological, Distress, Indian, Body image, Hair loss

## Abstract

**Background:**

Breast cancer is the commonest form of cancer among women globally, including in India. The rising incidence in the developing world is thought to be due to increased life expectancy, urbanisation, and adoption of western lifestyles. A recent systematic review found that Indian women living in India or as immigrants in Canada experienced a range of psychological distresses both ameliorated and exacerbated by cultural issues personally, within the family, within their community, and in the context of faith, and only two of the five qualitative studies explored the experience of women with breast cancer living in India. Distress may also affect treatment compliance.

**Aim:**

The aim of the study was to explore the psychological distresses experienced by Indian women with breast cancer living in Kerala, South India, during and after treatment and to understand better what helped to relieve or increase these distresses.

**Methods:**

In-depth interviews were conducted with 20 consenting women undergoing treatment for breast cancer. Purposive sampling was used to obtain maximum variation in sociodemographic and clinical characteristics. Interviews were verbatim transcribed, translated into English, and back-translated to Malayalam to ensure that the meaning had not been lost. English data were analysed using thematic frame work analysis and synthesised to provide a deeper understanding of the individuals’ experience.

**Results:**

Three major themes emerged from the data. The first major theme was ‘far-reaching psychological distress’. This included anxiety, guilt, anger, and depression in response to the disease and physical side effects of treatment and issues relating to body image, especially hair loss and sexuality. The second major theme was ‘getting on with life’. Women tried to make sense of the disease, by actively seeking information, the role of medical professionals, and their practical adaptations. Many found a new future and a new way to live normal. The third major theme was the ‘support system’ strongly based on family, friends, faith, and the community which affect them positively as well as negatively.

**Conclusion:**

Psychological concerns related to disease and treatment are common in Indian women with particular emphasis on body image issues associated with hair loss. Family and faith were key support systems for almost all the women, although it could also be the causes of distress.

**Electronic supplementary material:**

The online version of this article (10.1007/s00520-020-05756-8) contains supplementary material, which is available to authorized users.

## Background

Breast cancer is the most common form of cancer among women of all racial and ethnic groups in the developed and developing world. International Agency for Research on Cancer registry data show that 45% of newly diagnosed cases of breast cancer and 55% of breast cancer–related mortality occur in low- and middle-income countries. Survival in North America is over 80% but around 50% in low-income countries [1]. This reflects a lack of early detection programmes and inadequate resources for active treatment and supportive and palliative care [2].

Breast cancer is now the most common cancer in women in India [3]. Psychological distress is common due to the diagnosis, fear of relapse or death, body image issues, and treatment-related side effects [4]. A good understanding of the psychological needs, cultural expressions, and coping methods of such women is imperative in order to inform service provision, and public and policy discourse for Indian women with breast cancer whether in India or part of the world diaspora. Women with unmet cancer-related psychological needs are less likely to present with early disease, agree to—or complete—treatment, and access professional support. However, interventions and services, including palliative care services, to address psychological distress are available in many regions.

A recent systematic review [5] found that Indian women living in India or as immigrants in Canada experienced a range of psychological distresses both ameliorated and exacerbated by cultural issues personally, within the family, within their community, and in the context of faith. The cultural issues identified were similar for the women irrespective of the country studied. Furthermore, even in India treated by Indians, the women did not always receive culturally congruent care; assumptions are being made about culturally competent care for an individual. Despite a number of observational studies, only two [6, 7] of the five qualitative studies in the review were set in India (Karnataka and West Bengal). India, the second most populous country in the world, is also socially and culturally the most diverse in the world [8]. We report the first qualitative study of women with breast cancer from the culturally distinct state of Kerala. The aim was to explore their psychological distresses, and what influenced them, during and after treatment in the context of their cultural expression within the overall culture of India.

## Methods

### Study design

We conducted semi-structured one-to-one interviews taking the cultural context of South India into account. One-to-one interviews recognised that some women could find it difficult to talk about personal experiences in front of others due to the range of experience and cultural expectations.

We used thematic analysis [9] to enable women to be heard directly rather than through further interpretation, as their voices are less central in healthcare decision-making in the Indian culture. This approach also helped us to study a relatively homogeneous group whilst allowing for variation in experience within that group. Though data collection did not use a theoretical framework, a theoretical framework of ‘cultural distress’ [10] influenced our analysis.

### Ethics

Approval was given by the Ethics Committee and Scientific committee of Amrita Institute of Medical Sciences and Research Center prior to recruitment. The study was performed in accordance with the 1964 Declaration of Helsinki and its later amendments.

### Setting

The interviews were conducted in a tertiary hospital in the South Indian state of Kerala (October 2015 to January 2016).

### Sampling, participants, and consent

Eligible participants were consenting adult women being treated for breast cancer able to participate in an interview. They were purposively sampled for specific characteristics to gain variation in experience and allow examination of convergence and divergence. Informed consent was taken prior to interview, including for use of anonymised quotes.

### Data collection

Interviews were conducted in Malayalam (regional language) to obtain rich, comprehensive, and uninhibited stories and used a topic guide (Table 1) based on research team expertise and the literature. Emerging new topics were incorporated into future interviews. Sociodemographic data and levels of distress (National Comprehensive Cancer Network Distress Thermometer [11]) were collected. Participants were interviewed (SD) on their own or with the carer in attendance (non-participatory). Non-verbal observations were recorded (CV). The interviews (duration 30 to 45 min) were audio-recorded, verbatim transcribed, translated into English (SD), and back-translated (CV) to ensure fidelity to the participants’ experience. Data saturation was considered adequate when no new codes were seen, and convergent stories predominated [12].

**Table 1:** Interview guide

Prompts for focused interview	
• Could you tell me more about yourself and the reasons for coming to the hospital? • Could you tell me more about your experience after being diagnosed with cancer? • Could you tell more about how you feel after you came to know the diagnosis? • Could you tell more about treatment that you are receiving/received? ○ Tell me about your understanding of the treatment. ○ Practical difficulties ○ Side effects-radiotherapy ○ Surgery: how did you feel after having your breast removed? How has this affected your relationship with your partner/spouse? • Could you tell how do you feel about the treatment? • Could you tell how you view your life after the diagnosis? • Could you tell more about the hopes in your life and whether it has changed since diagnosis? • Could you tell more about your daily activities? • During your treatment period, were you mentally disturbed in any way? • Are you able to talk about it with anyone? • Could you tell more about how you have been coping with the current situation? • What resources have you drawn to cope with your suffering? • How did you cope with difficult times in your life before you had cancer? • Has having cancer changed how you cope with things in your life? • Has having cancer changed your role in family and expectations by other family members? • Does faith or spirituality play a part in your life? In what way? • Can you tell me more about that? • How do you feel about talking about this and the interview?	

Analysis in English allowed the non-Indian researchers (MJ, AH) to participate helping to address potential challenges in reflexivity (SD and CV were department staff members and both from the culture involved). MJ and AH are UK researchers.

### Data analysis

Interviews and field notes were checked for accuracy and anonymised. The NVivo 12 software was used to manage data. English data were analysed in the following steps: (i) data familiarisation by reading and re-reading; (ii) line-by-line coding; SD, CV, and MJ independently coded the initial interviews and agreed codes, whilst allowing new codes to present (iii) coding of all transcripts (SD); (iv) discussion (SD, MJ) to describe developing patterns of commonality (themes or convergence) (v) agreement of analytic themes through further discussion (SD, CV, MJ) ensuring distinct themes with consistent data [13].

## Results

Sociodemographic and clinical characteristics are described in Table 2. The 20 participants (median age 56 years; 42 to 74) had a median level of distress of 3 (0–8) (lower quartile 0, upper quartile 4) (0 = no distress and 10 = extreme distress). Most participants were accompanied by their family carer.

**Table 2:** Sociodemographic characteristics and clinical characteristics

Variable	Number (%)
Age median (range of age in years)	56 (42 to 74)
Education	
School level	10 (50%)
Graduate level	9 (45%)
Not known	1 (5%)
Economic status	
Middle class	19(95%)
Upper middle class	1 (5%)
Marital status	
Married	17 (85%)
Widowed	3 (15%)
Religion	
Hindu	16(80%)
Muslim	1 (5%)
Christian	3(15%)
Main carer	
Husband	16 (80%)
Son	2 (10%)
Daughter	1 (5%)
Daughter-in-law	1 (5%)
Carer lives with patient	
Yes	19 (95%)
No	1 (5%)
Patient aware of the diagnosis	
Yes	19 (95%)
No	1 (5%)
Carer aware of the diagnosis	
Yes	20 (100%)
No	0 (0%)
Stage of disease	
I A	1 (5)
II A	7 (35)
II B	3 (15)
III A	7 (35)
Not available	2 (10%)
Treatment modality	
Surgery + RT + CT	9 (45)
Surgery + RT	5 (25)
RT alone	1 (5)
RT + CT	3 (15)
Surgery + CT	9 (45)

*RT*, radiotherapy; *CT*, chemotherapy

Three themes were generated: (1) far-reaching psychological distress of patients to disease and treatment, (2) getting on with life, (3) influence of their support system. The major themes, subthemes, and codes are described in Supplementary Table 1, and Tables 3, 4, and 5 give representative quotes under each subtheme.

**Table 3:** Representative quotes under theme 1

Major themes	Subthemes	Quotes
Far-reaching psychological distress	Anxiety	‘In my family in all these years there is a history of heart attack, my father’s father had it. I was expecting that since I am a bit overweight (laughs) I would be getting that and I never thought that I would get cancer’ (Participant 17; aged 40–50) ‘I never thought it is going to be this, I was convinced that I will not get this disease, somebody mentioned the word carcinoma and I didn’t know that it meant this, I always thought the word was cancer, also there was a mention of malignancy in mammogram, I didn’t know that was cancer either. So when doctor told me the result, I said I didn’t understand. Then he explained that it is the first stage of cancer: then I was really shocked’ (Participant 7; aged 40–50) ‘My main thoughts were, my children are still young, I have a husband, I am the one who looks after everything in house … (Participant 14; aged 50–60) ‘But when chemotherapy started I had lots of issues, I was alone in house with nobody to help me as I am used to doing things on my own, my mind was always tensed thinking ‘how did this happen to me, ‘oh, it has happened to me’ and I keep thinking about other people dying. I am normally an anxious person and I worry about small things and I had problems with depression in past … (Participant 13; aged 60–70) ‘I keep worrying about whether the disease will come again. I listen to doctor talking on radio and they say it could come again; some doctors say after some time it could come again. Here also when I see other patients they say they had it before, and it has come back second time. But my test are negative in armpit so I am a bit relieved.’ (Participant 11; aged 40–50) ‘I haven’t had any contact since the diagnosis, mainly because I am too tired and fatigued to do it and my husband is accepting that. He doesn’t have any problem with that.’ (Participant 16; aged 40–50) ‘I have some problems with my bladder and have difficulties sitting down, so I can't clean myself properly, husband is still keen on having sex, so it is a problem, also if we don't have physical contact he might develop some prostate problems , so I am worried about that (Participant 12; aged 60–70)
	Depression	‘So, when I saw Dr X for the first time, I did not say anything, I was crying throughout the consultation. My husband did all the talking; I was sitting and crying there all the time’ (Participant 7; aged 40–50) ‘Of course when I was diagnosed with this disease- because all say this is a terrible disease- I started thinking that I am doomed for a life time that my near and dear ones will undergo great mental suffering, because it has always been me who stood as a pillar that supported our family. That caused me great pain. Even now I am in that pain’ (Participant 2; aged 60–70) ‘So, during chemotherapy, when I am alone in house I become upset thinking about lot of things and had problems with all the medications. I lost my appetite, I am not able to go out of the house, I started losing my hair. I became more and lower in mood’ (Participant 13; aged 60–70)
	Other psychological response	‘If I had come earlier the chemo would have been avoided, it had become 2 cm about 1.5 to 2cm, if I had come earlier maybe if it was less than 1cm... (Participant 17; aged 40–50) ‘I was also very angry initially, when I reached home after chemotherapy due the discomfort in my body I used to get very angry and I used to speak angrily with all my family. I used to answer back to my family members, so my husband was angry with me.’ (Participant 9; aged 50–60) ‘Like I am able to talk to you also openly, but normally I don't talk to anybody or I don't open up easily. I don't talk to others because they don't retain what we tell them, they interpret in their own way. They also exaggerate and speak to others like that. (Participant 14; aged 50–60) ‘I am glad that I talked to you as I realise that I am able to talk about my disease and answer the questions in detail. I didn’t realise that I would be able to talk like this. I am glad I did it’ (Participant 16; aged 40–50)
	Distress due to physical side effects of treatment	‘I lost a lot of hair, I had long and thick hair, so I was very sad when I lost my hair, my hair was not even grey, it was still black in spite of my age. Now it is coming back and I am getting more grey hair now. All people used to comment on my long black hair but now everything is gone. My daughter had got a wig for me, I used it a couple of times when I went to temple, but when I used it I felt strange, so mentally I was not comfortable in using that.’ (Participant 12; aged 60–70) ‘That disease might not be a good cancer and is likely to be risky, but I only have to be ashamed of hair loss, but in front of people they think that hair loss is an important indicator of disease severity (laughs). (Participant 17; aged 40–50) “If it is in temple then definitely there will be questions about why I have covered my head. Even people who know us would say ‘Oh you have lost all your hair’? Then we will have to explain everything. So, I don’t go to temple but pray in the house, prayer is the one that leads me forward.” (Participant 16; aged 40–50) ‘I had brought a wig, but you see I have a grandchild and I was worried what will the kids think If they see me wearing that and I wore it once but I felt uncomfortable wearing that in front of kids even though they didn’t say anything. So, I have not worn it since.’ (Participant 18; aged (50–60)) “I discussed it with doctor (not Dr Y) and he was saying ‘Oh you will lose all of it’ jokingly. He didn’t give importance to it (nervous laugh) I used to care for my hair a lot---- and I already had short hair –then--- I got used to it. Now I am upset that it is not growing back and every day I keep checking to see whether it is growing back.” (Participant 18; aged 50–60) ‘No, I was not upset about that, I am not young, I am now old, I also know that there are new methods to cover that. But I don’t need any of that, I am now old and I was not upset by that’ (Participant 10; aged 60–70) ‘I was a bit upset thinking about that but then realised if I have a disease then there is no other way and no point in keeping it. (Participant 15; aged 50–60) ‘I had total mastectomy and I am a bit upset about that, especially when sitting down there is some disfigurement, so I have to be very careful about appearance while sitting down.’ (Participant 12; aged 60–70)

**Table 4:** Representative quotes under theme 2

Major themes	Subthemes	Quotes
Getting on with life	Making sense of the disease	‘My understanding is that surgery has completely cured it and chemotherapy also helped in the removal, if anything is left behind then radiation is to destroy the cells by burning them.’ (Participant 12; aged 60–70)
	Actively seeking information	‘I read about things, I try to be bold and brave in the situation rather than thinking I have a terrible disease. I gather information as much as possible, now there are lot of printed material available which are good sources of information. I am not scared about reading and getting more knowledge, especially there are lot of misconceptions among people.’ (Participant 14; aged 50–60) ‘Prayer, studying, reading more about it, else I was in a shocked stage, but knowing that it is different from other cancers, I hope it is true, if it is detected in primary stage there is 98% chance of cure, the doctor also reassure me that good cure rate with surgery at early stage and results look positive.’ (Participant 17; aged 40–50)
	Role of medical professional	‘Also, the consulting doctors are a relief; Dr X and Dr Y are very good at explaining things and we can ask them anything. Some doctors we can't ask anything.’ (Participant 14; aged 50–60)
	Practical aspects	‘Because I was thinking that we would have to spend lot of money, then my son said, mother you should not think like that, money is not an issue, the important thing is to get recovered from the disease.’ (Participant 6; aged 50–60) ‘Initially doctor said I need 16 doses of chemotherapy and then because I had problems in travelling and my son couldn’t take time off work and come all the time, so we discussed it with doctor and changed it to eight.’ (Participant 10; aged 60–70)
	New way to live normal	‘No, it is more hobbies like looking after my plants and vegetables, cooking my favorite recipes, doing things around the house that I like, watch my favorite programs on TV. I try and do things I enjoy so that I don’t become upset thinking about my disease.’ (Participant 13; aged (60–70))
	New future	‘I pray that nobody else would get this disease, whatever we say that we are not upset; this disease is a great sorrow for everyone. So whenever anybody or my colleagues call me to talk about this and I ask them and I advise them that even when they see any small swelling, I ask them to check it out so that nobody develops into this disease.’ (Participant 16; aged 40–50)

**Table 5:** Representative quotes under theme 3

Major themes	Subthemes	Quotes
Support system	Family and friends	‘Family was very supportive; my sister in law has come from Abu Dhabi to look after me, so that she can accompany me while I go for radiotherapy. Sometimes my husband comes with me and some days she accompanies me, someday she stays at home to get my kids ready for college every day. Before that my husband’s sister who is in Bahrain, came over during chemotherapy and stayed for the entire time. They all have been very supportive.’ (Participant 20; aged 50–60) ‘My husband is finding it very difficult to cope, especially taking time away from work and we try and manage between ourselves especially my son and brothers; but they also have family and so can't spend lot of time with me. Also, my son has exams now, so it is difficult to take time off. But when I think about my husband I am a bit upset.’ (Participant 14; aged 50–60) ‘I didn’t know about this decision. Once I was in the theatre they discussed with my husband about it and he said to go ahead with reconstruction. But now I feel they shouldn’t have done the reconstruction as it is causing me more discomfort ...’ (Participant 3; aged 60–70)
	Faith	‘God has planned something for me and it will happen like that. God doesn't do anything without a plan, also if something happens to me then God will provide for my children. I have faith in God. I go to temples, take part in religious ceremonies, so I believe that God wouldn’t let anything bad happen to me.’ (Participant 14; aged 50–60) ‘I am able to face others and speak with confidence to others and to you because of my faith. I believe that the disease will be completely cured by treatment. I used to visit temples but I don’t do it anymore, mainly due to my hair. Muslims can wear purdah overhead but both Christians and Hindus people will notice if we cover our head.’ (Participant 16; aged 40–50) ‘I used to go to temple all the time even before I had this illness and I used to pray all the time. But now since the diagnosis my faith seems to have decreased. I light the lamp and pray but I don’t spend a long time chanting prayers as I used to do. I used to spend lots of time in prayer before the illness. For the past few days I have been thinking about that actually but I don’t know the reason for my change in behaviour.’ (Participant 3; aged 60–70) ‘Then since the gynecologist checked me I didn't think this was going to happen, else I would have been more cautious, I was also observing a penance as I was going to visit a sacred temple. So, I did not do breast self-examination during that time, but my doctor had checked me and said everything was clear.’ (Participant 12; aged (60–70))
	Community	“Not really harassing, but they will ask me, ‘Oh, you have cancer? What happened? Are you undergoing chemotherapy?’ Even if somebody is not undergoing chemotherapy treatment, they will say, she is. So, we get mentally disturbed.” (Participant 1; aged 70–80)

### Far-reaching psychological distress

Participants described a range of emotions including shock, disbelief, anger, guilt, anxiety, depression, social isolation, and fear of recurrence. Some women became emotional during the interview, talking about it was difficult, although valuable. Many were worried about the future of their children, who were ‘too young’ at diagnosis and ‘still in school’. The younger mothers in particular expressed distress at the fear of not being with them as they grow up.

They worried about the process and personal effects of cancer treatments. Fear of not coping with physical symptoms of the disease and treatment was significant, in relation to how symptoms would affect their ability to function. Household work tended to be the sole responsibility of women, so being physically unable to fulfil that role added to their distress. They feared overwhelming symptoms resulting in treatment discontinuance, aggravating worries about disease recurrence. This added to high levels of vigilance about any further symptoms, seeking the reassurance of ‘negative tests’. Women described how side effects of chemotherapy and radiotherapy were distressing. Women responded by staying at home leading to social isolation, which aggravated the situation. Changes in their bodies due to loss of hair and breast, lymphoedema, and hand swelling due to intravenous access were major concerns.

Hair loss was a cause of serious distress. Women in Kerala are renowned for and proud of their long, thick black hair as a symbol of beauty and femininity. Hair loss can be seen as a sign of severe disease. Loss of hair was shameful and prevented social and religious participation. Whilst artificial hair helped some, others said neither them nor their families could get used to it. Some clinicians failed to recognise the importance of hair loss and dismissed their concerns. With regards to mastectomy, some women felt that the removal of breast caused disfigurement and affected their body image, whereas others were not bothered as long as the disease was cured. Older women felt no need for breast reconstruction surgery—also more expensive than mastectomy.

In general, women experienced a decreased or no sexual desire after/during treatment, especially with hormonal treatment, with fatigue, reduced libido, vaginal dryness, and worries about infection. A few older women reported abstinence prior to the diagnosis due to already declining health. Despite the effect on sexual activity, many women reported that their relationship with husband was not adversely affected; however, a few felt an expectation to satisfy their partner’s desire despite their lack of interest. Differences in desire for sexual activity between husband and wife could cause problems sometimes compounded by incorrect health beliefs.

Most women experienced low mood and other depressive symptoms in response to what was often seen as a terminal diagnosis. A few had had a psychiatric review; one woman described how she cried throughout the mental health consultation. Another described how she had no desire to live any longer, and the realisation that she was the emotional and practical lynchpin of the family added to her distress. Some women felt guilty and regretful for not performing breast self-examination or presenting earlier for symptoms or attending for mammograms. Some women responded with anger and frustration at the diagnosis, physical symptoms during treatment, or their inability to do their routine physical activities; anger was sometimes directed at family members. Men in the family taking responsibility for housework did not seem to be considered, perhaps because of the women’s patriarchal upbringing.

Women found it difficult to talk about their problems with their immediate family or friends due to lack of opportunities or fear of being judged. There was a lot of stigma associated with a cancer diagnosis, and women were reluctant for this to become public knowledge. Women did not expect or feel able to talk about their experience freely in their daily lives, some commenting that the interview itself was therefore helpful.

### Getting on with life

Once they overcame the initial shock of diagnosis, women tried to make sense of the disease, by actively seeking information and with the help of medical professionals, and by making practical adaptations. Many found a new future and a new way to live normally, but some found this difficult. The cancer diagnosis caused significant financial worries to many due to costs of treatment, often shared across immediate family members. Often, the participants lived far away from the treatment centre and the treatment might have to be compromised to accommodate travel.

Most women understood the cancer treatment well, having had good explanations from their medical professionals, and felt that their treating doctors were supportive, helping them to come to terms with the diagnosis and accept the treatment. This was unexpected and welcomed compared with the previous experience of medical care for many. Women wished to understand more, to be better educated, and actively gathered information from medical professionals, reading books and leaflets, and watching television. One woman felt comforted in the knowledge that breast cancer had a better cure rate than some other types. Women felt that it was their duty to warn others, to create awareness to encourage early detection and prevent experience of distress and stigma. Some women developed more confidence and resorted to new hobbies, helping to cope with their illness. This helped them to move forward.

### Influence of their support system

Their support system, strongly based on family, friends, faith, and community, affected them positively and negatively.

In general, immediate family was very supportive: taking time off work and helping with physical work, accompanying them to hospital appointments, providing financial support. However, where family members were unable to increase support due to other commitments, tensions could arise. Some felt worried about their partners not coping and managing the household work in their absence and about the future of their children. The accepted patriarchal structure of the family caused serious concern for one woman; she was not involved in the decision for her to have breast reconstruction, leaving her unhappy.

Most women in the study practiced Hinduism, with Christianity and Islam also represented. Faith was a major source of support for most, helping the women to cope. Faith was a family affair, praying and reading spiritual books together. Some believed that God had enabled an early diagnosis allowing lumpectomy rather than mastectomy. However, loss of hair affected treasured observations of rituals greatly, with some women avoiding worship places especially churches and temple. However, in some, faith was seriously challenged, believing cancer to be a punishment from God, leading to bewilderment and sense of injustice. For others, faith was a barrier to accessing treatment resulting from ‘faith fatalism’ (God has already decided). There was a relatively common belief that cancer was a sign of God’s punishment, leading to stigma. Religious observance also could impact on other issues, for example, the belief that breast self-examination should not be done during the time of visiting sacred temple and that the examination is somehow wrong and unholy.

The immediate community appeared to increase rather than reduce the distress of the women. Most women had no one other than immediate family to talk to, and there were no support groups. In any case, they preferred not to talk about cancer to others because of the stigma; women felt under scrutiny and harassed. The ‘well-wishers’ who visited, despite good intentions, worsened their apprehension by asking questions, discussing misconceptions about the treatment.

## Discussion

This qualitative study, the first of its kind from a tertiary centre in Kerala, gives voice to the far-reaching psychological distresses faced by women undergoing treatment and the experiences which helped or hindered them through the process. Women experienced their diagnosis and treatment in the context of a prevailing societal attitude regarding the role of women as wife, mother, and the carer of older in-laws. Although the women in this study expressed distresses common to women from Western culture, the distress was amplified by this core expectation. Thus, a disease of a female organ and symbol of such womanhood, with treatments which further affected body image, sexual function, and ability to perform care of house and relatives, undermined a perceived primary purpose, even in more highly educated and employed women. However, despite the societal pressures, most women found their families and faith a source of strength, although some had unsympathetic or less flexible home situations. For some, faith was expressed unhelpfully, restricting access to self-care and acceptance of cancer treatments. Faith and faith practices also magnified the impact of some of the effects of treatment, such as loss of hair. Erroneous health beliefs and misinformation were commonly playing a role in delayed diagnosis, the psychological distress on learning of the diagnosis, and societal stigma associated with a disease that some thought could be contagious. Compassionate and honest information from professionals was greatly valued and helped ameliorate distress, but the women were not always able to participate fully in clinical decision-making. Support groups were lacking, leaving those without strong family support with little help.

Religious coping in response to illness can be both positive, providing comfort and reassurance, and negative, with painful spiritual struggle or doubt [14]. Most women in our study depended on faith to help them come to terms with the diagnosis and to cope. This is in keeping with previous studies showing that spiritual beliefs and practices are central to coping [15–19] especially in ethnic minorities [20]. However, some women considered their cancer as ‘God’s punishment’ which increased stigma and resulted in ‘faith fatalism’ which proved as a barrier to accessing treatment, consistent with previous studies [21, 22].

Women expressed anxiety, depression, fear of disease recurrence, worries about their children, and worries about change in role in family. This is similar to the distress experienced by all women where prevailing concerns include overall health, physical concerns, cancer recurrence or metastases, psychosocial concerns about children and burdening the family, and body image and sexual health concerns [20]. However, our findings indicate that these women experienced these concerns in the context of a perception that their *primary purpose* in society was as wife, mother, home-maker, and care-giver. Even for educated employed women in our study, and in Kerala—an Indian state viewed as affording higher status for women and known to have matrilineal descent among Nayar Hindus [23]—the primary distress was related to their inability to perform this role rather than loss of their employment. Traditionally, Indian women have a strong commitment to their immediate family, which includes their husband’s families, friends, and the religious community [24]. Family is usually considered more important than a career [25], and women’s wishes are expected to conform to family traditions, honour, and welfare [26]. Working women also bear the primary responsibility of child rearing [27]. Putting career ahead of family risks social censure, and there is pressure to conform [28]. However, this strong sense of family commitment was the most important support system for women.

Indian collectivist families promote interdependence rather than the ‘individualism’ of Western societies [29–31]. Indian women grow up in multi-generational households where the decision-making power rests with the male household members, initially fathers, then husbands, and then the adult son. Key decisions about treatment may be made by the oldest male relative or even by the whole family, but excluding the person being treated [31, 32]. Exclusion from decision-making, exacerbated by language barriers, was a cause of psychological distress of Indian women in Canada [21]. A recent Indian survey showed that most patients wanted full disclosure about their cancer even if prognosis was poor. In contrast, few of their family carers shared this information, believing it to be harmful [33]. As long ago as 1979, 83% of Indian women who underwent mastectomy wanted more information prior to surgery [34]. Although the women in our study largely had a good experience of their doctors, many Asian clinicians still believe that complete disclosure is undesirable and would increase patients’ distress [35]. Many Indian cancer patients are therefore unaware of the true nature of their cancer diagnosis [36]. In a survey of South Indian patients attending radiotherapy, only 62% were aware of their disease, of whom only 45% had been told by their doctors [37]. One of our patients in the study was unaware of the diagnosis, and the family member was present throughout the interview to ensure that she would not be ‘accidentally’ told by the interviewer.

Most participants raised body image issues due to loss of hair and of breast surgery. Alopecia is distressing and traumatic; a constant reminder of the disease itself [38–40]. Hair loss can lead to loss of self-confidence, low self-esteem, and heightened self-consciousness [41]. It is particularly difficult for Kerala women to cope with hair loss, as long hair is prised as a traditional feature of ‘Kerala-ness’ [42]. Most women saw hair loss as a barrier in accessing support including social or religious gatherings, and found artificial hair to be a poor substitute. However, despite this commonly acknowledged cultural issue, some clinicians failed to recognise how deeply hair loss affected women and were unsympathetic despite working within the same culture. Previous work has shown dissatisfaction with the doctor in 40% of patients, partly due to the doctor’s underestimation of the distress of hair loss [41].

Although some women felt that the removal of breast caused disfigurement, others were less concerned, perhaps feeling that this was most likely to effect a cure. A study among Gujarati-speaking Indian women living in the UK felt that their appearance did not matter as long as they are alive and healthy [43].

Most women felt that talking about their problems would be useful but did not have a ‘right person’ to talk to. They found that study participation provided an outlet for their thoughts. Indian women generally prefer to seek socio-culturally acceptable informal care [44], though this was not perceived to be helpful to the women in our study. Although most of the women had some psychological distress, few were referred to psychological services. Those that were found this useful. Referral rates for psychiatric/psychological services for cancer patients (even in institutions where such services are available) in both India and the West appear to be equally low [45].

### Strengths and limitations

As far as we know, this is the first qualitative study among breast cancer patients from Kerala State. However, due to the diverse nature of the culture of country, the data cannot be considered completely representative of the South Asian population. However, there were many similarities to the experiences of Indian women elsewhere, with added Kerala experiences to the picture. Most women interviewed were from the upper middle class, although some from more deprived strata were included.

### Clinical and research implications

Although not designed to be generalisable, these experiences resonate with the previous systematic review of Indian women in two other Indian states and Indian–Canadian migrants. There are important messages to clinicians caring for Indian women with breast cancer anywhere in the world. Clinicians must understand the prevailing societal attitude of women as wife, mother, and the carer of older in-laws, and the importance of hair loss. Even where the woman lives in another country, these values are often still in place. Women greatly appreciated compassionate information from professionals, but were not always able to participate in clinical decision-making. A family-centred approach to care, which includes the woman in decision-making, is needed. Culturally congruent support groups are needed. Faith is important for coping, and tailored chaplaincy services should be an integral part of clinical services and explored particularly for women refusing or with poor adherence to cancer treatments.

## Conclusions

Psychological concerns related to disease and treatment are common in Indian women in relation to prevailing societal attitudes regarding the role of women as wife, mother, and the carer of older in-laws. Hair loss caused particular distress. Family and faith were key support systems for almost all the women, although it was also the causes of distress for some.

## Electronic supplementary material

ESM 1 (DOCX 13 kb)

## Data Availability

All data has been presented in the manuscript and additional material as supplementary material
