# Supplementary material for: ‘I don’t talk about my distress to others; I feel that I have to suffer my problems...’ Voices of Indian women with breast cancer: a qualitative interview study
Source: Support Care Cancer. 2020 Sep 21;29(5):2591–600. doi: 10.1007/s00520-020-05756-8 (PMC7981292; doi:10.1007/s00520-020-05756-8)
Supplement: Supplementary file 1 — (DOCX 13 kb) [file 520_2020_5756_MOESM1_ESM.docx]

**Supplementary table : Major themes, subthemes and codes**

| **Major themes** | Sub themes | Codes |
| --- | --- | --- |
| **Far-reaching psychological distress** | Anxiety | Anxiety, fear, uncertainty, worries about future  Anxiety in relation to: Effect on function, Inability to do physical activity, Change in routine habits and Impact on sexual function.  Surprise, unexpected , |
|  | Depression | Sleep issues, Loss, grief  Lack of interest  Loss of hope |
|  | Other psychological response | Guilt, Blame, Shock, Anger, Inability to talk about problems  Distress due to physical side effects of treatment, Alopecia, scarring, skin changes, Lymphedema, Treatment experience, body image |
| **Getting on with life** | Making sense of the disease | Disease perception, understanding, knowledge by patient |
|  | Actively seeking information | Seeking knowledge  Utilisation of resources |
|  | Role of medical professional | Role of medical professionals |
|  | Practical aspects | Access to care, transport  Economic, Financial  lack of resources |
|  | New way to live normal | Rationalisation  Information Intellectualization  Pragmatism, stoicism, acceptance  Positive thinking  Maintaining normal  Practical, active, adaptation, personal life style change  Keeping busy |
|  | New future | Responsibility for people  Action: warn others  Raising awareness  Positive thinking: Resilience  Caring for self, hobbies  Emotional resilience  Facing up to the future  Downwards comparison |
| **Support system** | Family and friends | Role of family in decision making  Relationship with husband  Family changes, role in family  Feeling of burden to family  Role of friends |
|  | Faith | Prayer  Faith, spirituality, faith fatalism  Bargaining with God |
|  | Community | Perception by community  Lack of empathy from professional  Social stigma  Social isolation  Invasion of privacy  Preference of female doctor |
